# Childhood Trauma and Adult Loneliness as Predictors of Sleep Health in Blackfeet American Indian Adults

**DOI:** 10.1007/s12529-025-10420-8

**Published:** 2026-02-11

**Authors:** Mia Hutchison, Betty Henderson-Matthews, Zachary J. Wood, Reece Kothe, George Heavy Runner, Lester Johnson III, Mary Ellen Lafromboise, Melveena Malatare, Emily Salois, Jason R. Carter, Peter Helm, Neha A. John-Henderson

**Affiliations:** 1 Montana State University, Bozeman, United States; 2 Blackfeet Community College, Browning, United States; 3 Montana State University, Center for American Indian and Rural Health Excellence, Bozeman, United States; 4 Baylor University, Waco, United States; 5 Browning, MT USA

**Keywords:** Childhood trauma, Loneliness, Sleep health, American Indian adults

## Abstract

**Background:**

Prior work links childhood trauma and adult loneliness to sleep health in adulthood in Blackfeet American Indian adults. In the current study, in a sample of 275 Blackfeet American Indian adults, we investigated whether the relationship between childhood traumatic events and adult sleep health is moderated by reported levels of loneliness in adulthood. We hypothesized that low levels of adult loneliness may offset the negative impact of childhood trauma exposure on sleep health in adulthood.

**Method:**

The study utilizes Community Based Participatory Research methods and approaches and is based on a long-standing research partnership between faculty at Montana State University and Blackfeet Community College.

**Results:**

Using a linear regression, controlling for age, gender, and education, we found that adult loneliness was a significant moderator of the relationship between childhood trauma and sleep health in adulthood (B = -0.19, t(264) = -3.13, *p* = 0.002). Simple slopes analyses indicated that the relationship between high levels of childhood trauma and poor sleep health was only evident for individuals who also reported high levels of adult loneliness.

**Conclusion:**

The current findings suggest that interventions targeting adult loneliness may reduce the risk for poor sleep health typically associated with high levels of childhood trauma.

## Introduction

Childhood trauma is generally defined as experiences during childhood that involve exposure to harm, including abuse, neglect, violence, or significant loss, which can have lasting impacts on a child's emotional, psychological, and physical well-being [1]. As childhood is a critical period for brain development and emotional growth, trauma during this period can shape an individual's perceptions, coping mechanisms, and relationships [2].

American Indian communities have disproportionately high levels of childhood trauma [3]. One study revealed that American Indian populations reported the highest incidence of adverse childhood experiences compared to any other racial or ethnic groups [4]. This research also found that Native American children were nearly twice as likely to experience or witness violence in their communities, live with caregivers struggling with substance abuse, or witness domestic violence between parents.

The high incidence of childhood trauma in American Indians is linked to the historical trauma and loss they have endured [3]. American Indians were subject to U.S. policies such as the forced assimilation of Native children into boarding schools which involved forced separation from their families, loss of their tradition, language, and practices. Many children in these boarding schools were subjected to physical, sexual and emotional abuse. Such traumatic experiences affected not only the individuals in these schools and their own relationships but also the parent–child relationships of subsequent generations [5]. More generally, American Indian historical loss and trauma have contributed to a high incidence of depression, substance abuse, and PTSD within Native communities [5, 6].

Childhood trauma has been linked to negative sleep health across racial and ethnic groups [7]. For instance, a longitudinal study examining the relationship between childhood trauma and poor sleep health in adulthood found that higher reported childhood trauma was associated with lower sleep health scores in both diary-based and actigraphy-based assessments [8]. One possible link between childhood trauma and negative sleep health is social isolation or conversely social connectedness. Prior research indicates that social factors may be particularly relevant informants of sleep for American Indian adults who experienced high levels of childhood trauma. Specifically, following the onset of the COVID-19 pandemic and the associated mandates for social distancing and isolation, American Indian adults with high levels of childhood trauma reported the greatest declines in sleep quality [9]. It is also important to acknowledge that childhood trauma can have significant effects on an individual’s social relationships and their willingness to connect with others [10]. Specifically, individuals who have experienced trauma during childhood may struggle with trust, emotional intimacy, and communication in relationships, often leading to challenges in forming healthy, supportive connections in adulthood [11].

Loneliness is the distressing feeling that arises when individuals perceive their social connections and relationships as insufficient [12]. Research has consistently shown that loneliness is linked to poorer sleep outcomes. Specifically, individuals who experience loneliness tend to report worse sleep quality compared to individuals who are satisfied with their social connectedness (i.e. less lonely). A longitudinal study examining twins found that when one twin felt lonelier than the other, they were more likely to experience disrupted sleep [13].

In recent years, there is increasing evidence supporting a relationship between loneliness and sleep in American Indian adults. For example, one study found that American Indian adults who felt lonelier on a given day experienced higher wake after sleep onset (WASO) that night compared to their average WASO over a week-long period. This suggests that loneliness may play a significant role in disrupting sleep for American Indian adults. In addition, the study controlled for factors such as anxiety, depressive symptoms, and adverse childhood experiences, and found that loneliness continued to predict WASO above and beyond these factors [14].

Sleep health in American Indians is a highly relevant health outcome that could contribute to enduring health inequities given that a robust literature indicates that poor sleep is implicated in the chronic conditions that disproportionately affect American Indians [15, 16]. Furthermore, prior work indicates that compared to other racial and ethnic groups, American Indians have worse sleep, including shorter sleep duration [17]. As such, research is needed to understand the factors that relate to sleep health in this population to inform future efforts to improve sleep health and consequently reduce existing inequities in health.

The Blackfeet nation in Northwest Montana is currently 1.5 million acres in size and has approximately 15,560 enrolled members. Based on the 2022 United States census, the Blackfeet Nation has a population density of 4.4 people per square mile. In comparison, the national average is 93.29 people per square mile, while its average is 8 people per square mile [18]. Given the low population density, Blackfeet community members may experience high levels of isolation, and thus loneliness may be a particularly relevant informant of health outcomes including sleep health.

In prior research in the Blackfeet Community, social connectedness offset the typically observed relationship between childhood trauma and levels of immune system inflammation in adulthood [19]. Based on these findings highlighting social connectedness as a resilience factor, the current study aims to examine whether the link between childhood trauma and sleep health is moderated by adult levels of loneliness in Blackfeet American Indian adults. It is hypothesized that, among individuals with high levels of childhood trauma, those who also report low levels of loneliness in adulthood will experience better sleep health compared to those with similarly high levels of childhood trauma who report high levels of loneliness in adulthood.

## Methods

The current data is from an ongoing Community-Based Participatory Research Project led by investigators at Montana State University and Blackfeet Community College. This partnership and project are rooted in many years of collaboration and research studies focused on social connectedness and health [17, 20]. The current project is named *Aa Koo Moo Waap* (AKMW) which can be translated to People Coming Together. The overarching goal of AKMW is to understand the long-term relationships between multiple indices of social connectedness and health-relevant outcomes (cardiometabolic health, mental health, and sleep health). Data for AKMW is collected over a 2-year period, and outcomes are measured at 3 time points. The current manuscript uses data from the baseline data collection. The study was approved by the Blackfeet Institutional Review Board and all participants provided written informed consent before participation.

Eligibility criteria included being between the age of 18–65, self-identification as American Indian and eligible participants had to reside in the Blackfeet Nation at the time of data collection. Once eligibility was confirmed, participants were scheduled to come to Blackfeet Community College for data collection. Data collection took place in April of 2023. Our goal was to recruit 280 participants based on a power analysis indicating that this sample size would be adequate to conduct planned longitudinal analyses and to account for potential attrition. The research team advertised across the community using established connections, word of mouth, and flyers. The research team made a special effort to recruit participants from all communities across the Blackfeetreservation. Some participants resided close by to the community college while others traveled up to 1 h to participate. For participants traveling from far distances, we made sure to schedule their appointments for data collection to provide sufficient time for travel. Our final sample included 276 Blackfeet American Indian adults.

All data was collected in-person at the Math and Sciences Building of the Blackfeet community College. Participants had the option to complete a survey using Qualtrics' online platform on desktop computers or by using paper and pencil. The research team consisted of faculty and students from Montana State University and a team of 20 Blackfeet community members including Blackfeet community college staff, students and faculty, and nurses. Anthropomorphic measures, blood pressure, and blood samples were collected by Blackfeet community members.

### Analytic Plan

All statistical analyses were conducted using IBM SPSS Statistics (Version 30). We used a linear regression to investigate whether loneliness in adulthood moderates the relationship between childhood trauma and adult sleep health. The data met the key assumptions of ordinary least squares regression, with normality of residuals, homoscedasticity no evidence of multicollinearity or autocorrelation. There were four participants who were missing data for the variables included in our analyses. As a result, these four participants were not included in the reported analyses and results.

All variables were mean-centered. In Step 1 of the regression, we entered covariates including biological sex, age, highest level of education, and current symptoms of anxiety and depression. In Step 2 we added childhood trauma; in Step 3 we added loneliness; and in Step 4 we added the interaction term for childhood trauma and loneliness.

### Measures

#### Covariates

Based on documented relationships between age, gender, education, symptoms of depression and anxiety and our outcome of interest, sleep health, we included these variables as covariates in our analyses [21–24]. While there are many environmental, behavioral and psychological variables related to sleep health, we chose to include these demographic variables and mental health indices given the robust literature linking these variables to sleep health across racial and ethnic groups [21–24]. Age and gender were self-reported and the highest level of education was reported using the following categories: 1 (Did not complete high school), 2 (High school diploma or GED), 3 (Some college but no degree), 4 (associate's degree), 5 (bachelor’s degree), 6 (master’s degree or higher). Symptoms of depression and anxiety were measured using the Hospital Anxiety and Depression scale [25]. The HADS includes 14 items, seven of which focus on measuring anxiety symptoms and seven items focusing on depressive symptoms. Items are scored on a 4-point scale, 0–3, with higher scores reflecting more symptoms of depression and anxiety. In the current sample, the HADS demonstrated good reliability for the anxiety subscale (Cronbach alpha = 0.83), and acceptable reliability for the depressive symptom subscale (Cronbach alpha = 0.67).

#### Childhood Traumatic Events Scale

As a measure of childhood trauma exposure participants completed the Childhood Traumatic Events Scale (CTES) [26]. This 6-item scale measures both exposure to and perceived impact of childhood traumatic events prior to age 17. Questions ask about the death of a family member or very close friend, major upheaval between parents, traumatic sexual experience, violence, extreme illness or injury and any other major upheavals. If the participant indicated they experienced an event, the response was coded as 1, and if they did not experience the event, the response was coded as 0. As a measure of the total number of childhood traumatic events, we summed responses to each of the events in the scale. The possible range for this measure is 0–6 with higher scores reflecting more childhood traumatic events.

#### Loneliness

As a measure of loneliness, participants completed the Short Loneliness Scale (LON) [27]. The scale consists of the following items: “How often do you feel that you lack companionship?” “How often do you feel left out?” and “How often do you feel isolated from others?” Participants respond to the three items using a 4-point rating scale (1 = *never,* 2 = *once in a while,* 3 = *fairly often,* and 4 = *very often*). Scores from the three items are summed with higher scores reflecting more loneliness (possible range 4–12). In the current sample, the scale had good reliability (Cronbach alpha = 0.88). This scale has been used to measure loneliness and its relation to sleep in American Indian adults [14].

#### Sleep Health

We used the 6-item RU-SATED questionnaire as a measure of sleep health [28]. This scale has been used previously as a measure of sleep health in Blackfeet American Indian adults [14, 29]. The scale demonstrated acceptable internal reliability in this sample (Cronbach alpha = 0.70). The measure consists of 6 questions to measure dimensions of sleep that are frequently related to health outcomes. These dimensions include regularity, satisfaction, alertness, timing, efficiency and duration and are measured with the following questions: “Do you get in and out of bed at similar times each day?” (regularity); “Are you satisfied with you sleep?” (satisfaction); “Do you stay awake all day without dozing?” (alertness); “Are you asleep (or trying to sleep) between 2:00 AM and 4:00 AM?” (timing); “Do you spend less than 30 min awake at night (This includes the time it takes to fall asleep and awakenings during the night)?” (efficiency); and “Do you sleep between 6 and 8 h per day?” (duration). Answer choices for all questions are *Rarely/Never* (0), *Sometimes* (1), and *Usually/Always* (2). Answers are summed to create a global measure of sleep health (possible range 0–12), with higher numbers reflecting better sleep health.

## Results

Descriptive statistics and bivariate correlations are reported in Table 1. The median age in the sample was 37, which is higher than the median age of 30.4 observed in the Blackfeet community in the American Community Survey (ACS) 5-year estimates [30]. The age range of this sample was 18–65, and according to the ACS 5-year estimates, 57% of community members are between the ages of 18 and 64 [30]. The current sample was 64% female, while the ACS reported that 52% of Blackfeet community members were female [30] indicating that females were overrepresented in the current sample. In the present sample, 15.6% did not complete high school, 30.5% reported that their highest level of education was high school, 21.8% reported that they completed some college but did not obtain a degree, 16.0% reported that an associate’s degree was their highest level of education, 11.6% reported obtaining a bachelor’s degree, and 4.4% reported obtaining a master’s degree or higher. According to the ACS 5-year estimates the highest level of education was high school for 31% of respondents from the community [30], similar to the 30.5% observed in the current sample.

**Table 1:** Descriptive statistics and bivariate correlations

Variable	M	SD	1	2	3	4	5	6	7	8
1. Age	38.41	14..41	-	.081	.169**	-.197**	-.070	-.021	-.032	.048
2. Biological Sex	64.4% Female			-	.191**	.118	-.023	.180**	.111	.096
3. Education	30.5% High school diploma or GED				-	-.077	.178**	.153*	.016	.268**
4. Anxiety Symptoms	8.96	4.36				-	.653**	.261**	.449**	-.242**
5. Depression Symptoms	5.66	3.33					-	.156**	.448***	-.291**
6. Childhood Trauma Exposure	2.30	1.57						-	.321**	-.032
7. Loneliness	5.85	2.40							-	-.126*
8. Sleep Health	7.24	2.51								-

**Fig. 1:**
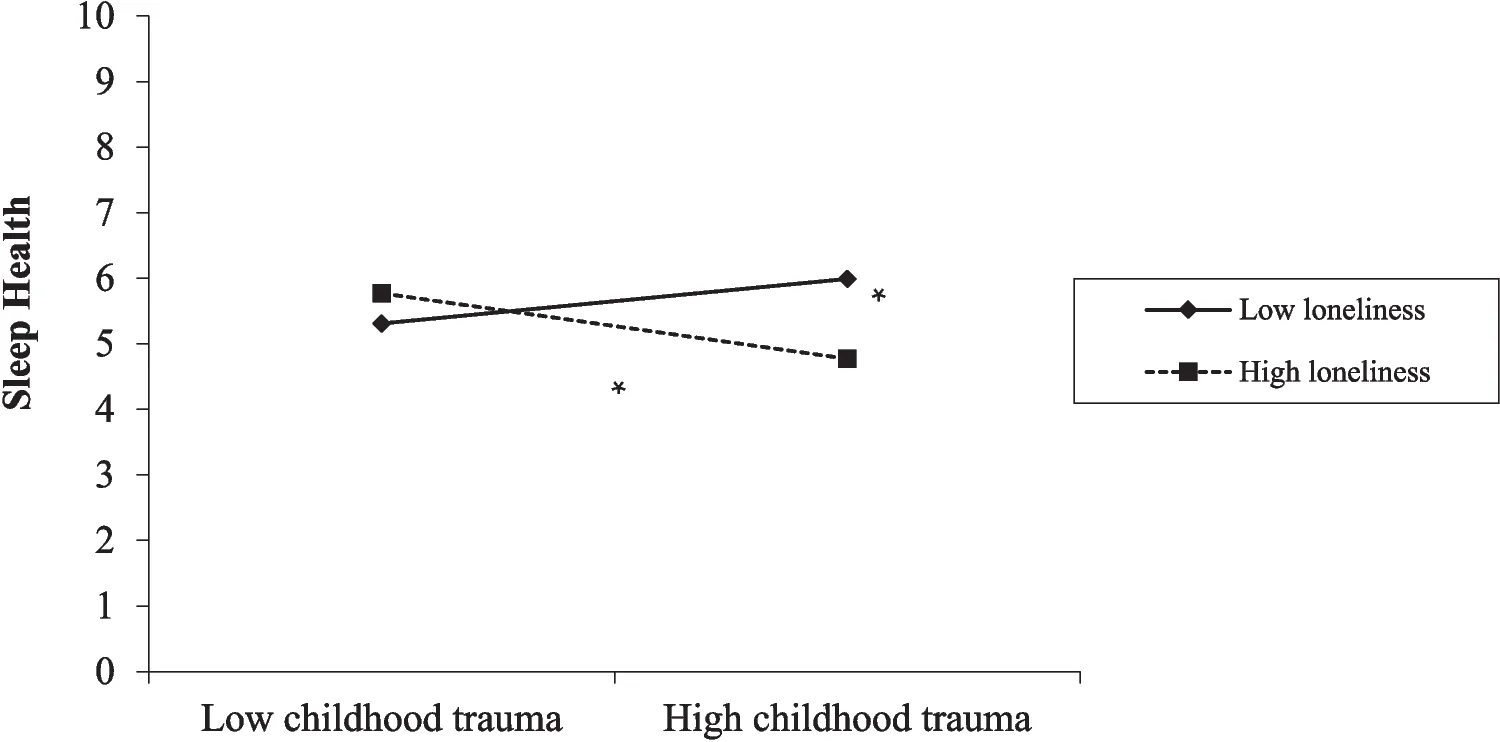
Results of a hierarchical linear regression with childhood trauma and adult loneliness predicting sleep health. Asterisks in the graph represent statistical significance at *p* <.05

The interaction between childhood trauma and loneliness was a significant predictor of sleep health (B = −0.19, t(264) = −3.13, *p* = 0.002). The full results for this regression model are reported in Table 2 and the pattern of the interaction is displayed in Fig. 1. Further probing of the interaction using simple slopes analyses showed that the relationship between childhood trauma and sleep health was only evident at high levels of loneliness (1 SD above the sample mean) (b = −0.52, *t*(264) = −2.54, *p* = 0.01). In addition, the relationship between loneliness and sleep health was only evident in individuals with high levels of childhood trauma (1 SD above the sample mean) (b = −0.64, t(264) = −3.28, *p* = 0.001).According to this criteria, in this sample there were 19 participants who were high in both childhood trauma and loneliness. Utilizing the Johnson-Neyman technique [31], we identified regions of statistical significance for the interaction between childhood trauma and loneliness in predicting sleep health. In the current sample, the relationship between childhood trauma and sleep health was significant for individuals who had a loneliness score of 7 or higher (possible range (4–12)). Our analyses indicated that 19.04% of the sample had loneliness at or above this level. Separate Johnson-Neyman analyses indicated that the relationship between loneliness and sleep health was statistically significant for participants who had a childhood trauma score of 4 or above (possible range 0–6). In the current sample, 10.25% of the sample had a childhood trauma score at or above this level.

**Table 2:** Hierarchical linear regression with childhood trauma and loneliness predicting sleep health. Note: Gender was coded as Male = 0, Female = 1

Step	Variable	Unstand *ß*	Coeff Std. Error		Stand *ß*	*t*	Sig	R^2^,R^2 change^
1	Age	−0.01	0.01		−0.03	−0.54	0.59	
	Gender	0.31	0.27		0.07	1.18	0.24	
	Education	0.39	0.11		0.22	3.66	< 0.001	
	Anxiety Symptoms	−0.08	0.05		−0.13	−1.67	0.10	
	Depressive Symptoms	−0.12	0.06		−0.16	−2.14	0.03	
								.14,.14
2	Age	−0.00	0.01		−0.03	−0.55	0.59	
	Gender	0.33	0.27		0.07	1.21	0.23	
	Education	0.40	0.11		0.22	3.66	< 0.01	
	Anxiety Symptoms	−0.07	0.05		−0.13	−1.59	0.12	
	Depressive Symptoms	−0.12	0.06		−0.17	−2.13	0.03	
	Childhood Trauma	−0.05	0.15		−0.02	−0.33	0.74	
								.57,.42
3	Age	−0.01		0.01	−0.03	−0.54	0.59	
	Gender	0.33		0.27	0.07	1.21	0.23	
	Education	0.40		0.11	0.22	3.65	<.001	
	Anxiety Symptoms	−0.07		0.05	−0.13	−1.54	0.12	
	Depressive Symptoms	−0.12		0.06	−0.16	−2.04	0.04	
	Childhood Trauma	−0.05		0.16	−0.02	−0.31	0.75	
	Loneliness	−0.01		0.17	−0.01	−0.06	.95	.57,.00
4	Age	−0.01		0.01	−0.01	−0.14	0.89	
	Gender	0.36		0.23	0.08	1.35	0.18	
	Education	0.35		0.11	0.20	3.20	< 0.01	
	Anxiety Symptoms	−0.06		0.05	−0.11	−1.34	0.18	
	Depressive Symptoms	−0.14		0.06	−0.19	−2.34	0.02	
	Childhood Trauma	−0.02		0.15	−0.01	−0.15	-.88	
	Loneliness	0.13		0.17	0.05	0.73	0..47	
	Childhood Trauma x Loneliness	−0.44		0.14	−0.19	−3.13	< 0.01	.59,.02

## Discussion

To our knowledge, this study is the first to investigate whether adult levels of loneliness moderate the relationship between childhood traumatic events and sleep quality in adulthood. The relationship between childhood trauma and sleep has been documented across racial and ethnic groups [7] and in American Indian adults [9]. In general, the onset of the COVID-19 pandemic negatively affected the sleep quality of Americans [32]. Specific to American Indian adults, one study found that declines in sleep quality following the onset of the pandemic were particularly pronounced for those individuals with high levels of childhood trauma [9]. However, this study did not investigate whether loneliness or social connectedness more generally moderated the relationship between childhood trauma and changes in sleep quality following a life event.

In the current study, the data provide preliminary evidence that social connectedness moderates the relationship between childhood trauma and sleep health in a sample of Blackfeet American Indian adults. As hypothesized, this relationship between childhood trauma and sleep health was only evident at high levels of loneliness. In addition, the relationship between loneliness and sleep health was only evident in individuals with high levels of childhood trauma. The relationship between loneliness and sleep health was not significant for individuals with low levels of childhood trauma. The findings provide preliminary evidence to support the notion that satisfaction with one’s level of social connectedness (i.e. low levels of loneliness) may offset the risk for sleep health typically associated with high levels of childhood trauma.

Several factors may help explain the current findings. First, loneliness is often inversely related to social support; individuals who are lonely tend to isolate themselves more, resulting in less social support, while those with stronger social networks report feeling less lonely [33]. Social support has been shown to play a crucial role in moderating the negative impacts of childhood maltreatment on emotional processing, particularly in relation to negative emotions [34]. When an individual experiences significant loneliness, they may lack the emotional and psychological resources needed to cope with stress. As a result, this lack of support could hinder efforts to cope with the consequences of childhood trauma, contributing to conditions such as increased stress, hyperarousal, and difficulty regulating emotions. These factors can disrupt sleep patterns and have a negative impact on sleep health. Another possible explanation for the moderating role of adult loneliness on the association between childhood trauma and sleep health is that both childhood trauma and loneliness are linked to increased levels of stress and anxiety [1]. Trauma is believed to induce hyperarousal, a state of increased alertness and stress that disrupts sleep [34]. Loneliness could exacerbate this effect, as individuals who are lonely might feel more anxious or vulnerable, further disrupting their ability to relax and fall asleep.

Since this study is cross-sectional, inferring causation is not possible. There could be other variables related to loneliness that may affect sleep that were not considered in the current study. While the current analyses accounted for demographic variables and indices of mental health that have been linked to sleep health across racial and ethnic groups [21–24], there are many factors that affect sleep health that are not accounted for in the current analyses. In future work it will be important to further examine environmental, behavioral and psychological factors that may also affect sleep health.

In addition, correlations may be confounded by bidirectionality. For example, poor sleep health could lead to greater loneliness, while loneliness may also contribute to sleep disturbances. Future research could address this by measuring these constructs daily to elucidate the directionality of the relationship. At the completion of *Aa Koo Moo Waap,* we will be able to investigate whether childhood trauma and loneliness interact to predict longitudinal changes in sleep health.

It is also important to acknowledge that limited access to transportation or costs associated with travel may have deterred participation in this study for individuals from communities on the reservation that are not near the community college. Although we made special efforts to offset obstacles to traveling for these individuals, it is possible that representation from these communities in the current sample may be limited due to these obstacles.

The current findings suggest that efforts to reduce loneliness in Blackfeet American Indian adults with high levels of childhood trauma could improve sleep health. Childhood trauma often leads to long-term emotional and psychological challenges, such as feelings of isolation and loneliness, which can worsen sleep problems and lead to poor sleep quality. In American Indian communities, these issues may be compounded by cultural, social, and historical factors, including systemic trauma and marginalization. Addressing loneliness could help alleviate these feelings of isolation, ultimately improving sleep health in Blackfeet American Indian adults.

As noted previously, understanding the psychosocial factors that affect sleep health is of great relevance to the Blackfeet community and observed health inequities given that sleep is implicated in the chronic conditions that disproportionately impact Blackfeet community members [15, 16]. Furthermore, research on sleep health and its predictors is warranted in American Indian communities given its link to suicide and substance misuse [35, 36], both of which are observed at high rates in these communities [37, 38]. Future interventions focused on improving sleep health for community members by targeting loneliness could not only improve sleep health but could also address enduring disparities in health-relevant outcomes for Blackfeet American Indian adults.

## Data Availability

Requests to access primary data must be sent to the Blackfeet Institutional Review Board at bnirbnaturalsci@bfcc.edu.

## Declarations

Informed consent was obtained from all individual participants included in the study.

All procedures performed in studies involving human participants were in accordance with the ethical standards of the institutional and/or national research committee and with the 1964 Helsinki declaration and its later amendments or comparable ethical standards.
